# Nature Inspired MIMO Antenna System for Future mmWave Technologies

**DOI:** 10.3390/mi11121083

**Published:** 2020-12-07

**Authors:** Saifur Rahman, Xin-cheng Ren, Ahsan Altaf, Muhammad Irfan, Mujeeb Abdullah, Fazal Muhammad, Muhammad Rizwan Anjum, Salim Nasar Faraj Mursal, Fahad Salem AlKahtani

**Affiliations:** 1 Electrical Engineering Department, College of Engineering, Najran University, Najran 61441, Saudi Arabia; snmursal@nu.edu.sa (S.N.F.M.); fsalkahtani@nu.edu.sa (F.S.A.); 2 School of Physics and Electronic Information, Yanan University, Yanan 761000, China; 3 Electrical Engineering Department, Istanbul Medipol University, 34083 Istanbul, Turkey; 4 Department of Computer Science, Bacha Khan University, Charsadda 24420, Pakistan; mujeeb.abdullah@gmail.com; 5 Electrical Engineering Department, University of Engineering and Technology, Mardan 23200, Pakistan; 6 Department of Electronic Engineering, The Islamia University of Bahawalpur, Bahawalpur 63100, Pakistan; engr.rizwan@iub.edu.pk

**Keywords:** directivity, Envelope Correlation Coefficient (ECC), gain, Multiple Input Multiple Output (MIMO), mmWave, nature inspired

## Abstract

In this work, a new Multiple Input Multiple Output (MIMO) antenna system with a novel shape inspired by nature is proposed for Fifth-Generation (5G) communication systems. The antenna is designed on a Rogers 5880. The dielectric constant of the substrate is 2.2, and the loss tangent is assumed to be 0.0009. The gain of the system for the desired bandwidth is nearly 8 dB. The simulated and the measured efficiency of the proposed system is 95% and 80%, respectively. To demonstrate the capability of the system as a potential candidate for future 5G communication devices, MIMO key performance parameters such as the Envelope Correlation Coefficient (ECC) and Diversity Gain (DG) are computed. It is found that the proposed system has low ECC, constant DG, and high efficiency for the desired bandwidth.

## 1. Introduction

The mobile communication systems’ evolution is progressing through the stage where data rates in the range of multiple gigabits per second are required. The vast need for higher data rates is challenging for researchers, while the solution that has emerged is to move towards the higher portion of the spectrum, i.e., mmWave, above the 6 GHz frequency band [1,2,3]. Thus, the already congested spectrum (below 6 GHz) has to be left behind, and the new mmWave spectrum providing a huge bandwidth has to be utilized [4,5]. Feasibility studies have been done to see whether this higher frequency range is capable of delivering the required data rates or not [6]. According to the experimental results, communication based on mmWave, above 6 GHz, is suitable in order to become a standard for the future [7]. There are several frequency bands that have been allocated for mmWave communication including licensed and unlicensed bands [8,9,10]. For example, the 28 and 38 GHz frequency band are licensed, while the 57–64 GHz and 164–200 GHz frequency bands are unlicensed; however, the unlicensed bands suffer from high atmospheric attenuation per kilometre [11,12,13]. To operate efficiently in these frequency bands, the antenna needs to possess high gain and bandwidth or Multiple Input Multiple Output (MIMO) features [14,15]. In the literature [16,17,18,19,20,21,22,23,24,25], several antennas have been reported for mmWave applications. Reference [17] reported a circular polarized MIMO antenna with four ports. The metamaterial surface is surrounded by the antenna elements to achieve better radiation characteristics, but the use of parasitic elements makes the MIMO design complex. Similarly, a 5G MIMO antenna operating at a wide frequency band of 23 to 40 GHz was presented with a total size of 81 × 80 mm${}^{2}$ [18]. Although the antenna covers a huge bandwidth and possesses MIMO features, these are at the cost of the large size. Reference [19] presented a dual band antenna with an array configuration, covering the frequency band between 28 and 38 GHz. The reported antenna achieves a high gain, but it does not possess a MIMO feature. Likewise, a single antenna with an overall size of 10 × 6 mm${}^{2}$ was proposed with a maximum measured gain of 6.59 dB [20]. Furthermore, two different types of metamaterial surface were employed, which make the practical realization of the reported work difficult. An integrated 4G/5G antenna covering the mmWave frequency band of 28 GHz with an overall size of 75 × 85 mm${}^{2}$ was reported [21]. The gain achieved in the mmWave band was 5.13 dB, while many side and back lobes were observed in the radiation patterns, which made the proposed design undesirable for mmWave communication. Reference [22] presented an 8 × 8 MIMO antenna with an overall volume of 31.2 × 31.2 × 1.57 mm${}^{3}$, resonating at the central frequency of 25.2 GHz with a peak gain of 8.732 dB, while the proposed antenna also suffered from many side and back lobes. Similarly, a mmWave antenna design with a T-shaped structure covering the frequency band from approximately 25.1 to 37.5 GHz was proposed in [23], while a MIMO antenna operating in the frequency band of 28 GHz [24] and a four port antenna with a size of 30 × 35 mm${}^{2}$ with a peak gain of 8.3 dB have been presented [25]. According to the above literature review, the reported antennas for mmWave applications have either a complex or a large structure. Furthermore, some of them achieve a satisfactory impedance bandwidth and gain, but they lack the MIMO feature.

In this paper, a nature inspired flower shaped MIMO antenna system is proposed. The antenna element is made up of five circular petals and a circular hub. These petals and hub are connected to a 50 ohm transmission line. The antenna operates in the mmWave frequency band. The design and operation characteristics of the proposed system make it a potential candidate for the upcoming mmWave technologies. This work is divided into different sections. Section 2 discusses the antenna design and its evolution to a MIMO system. Section 3 presents simulated and measured results for the proposed system. In Section 4, the MIMO performance parameters are analysed, while Section 5 presents a brief literature review and a summary of this work.

## 2. Antenna Design

**Figure 1:**
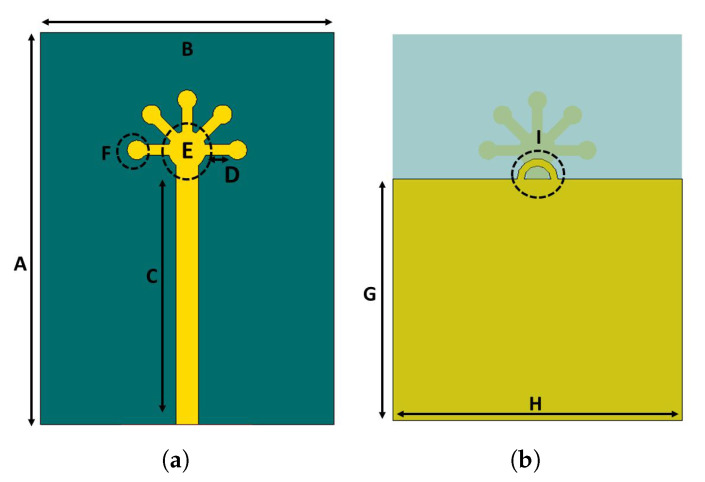
Proposed mmWave antenna: (**a**) front view; (**b**) back view.

The proposed MIMO antenna system is illustrated in Figure 1. The system is designed on a Rogers-5880. This substrate was chosen for two reasons: first, the low loss and, secondly, to be consistent with the research published by various researchers. The thickness of the substrate is 0.787 mm, and the permittivity of the substrate is 2.2. It has a loss tangent of 0.0009. The thickness of the ground plane and the radiating elements is 0.035 mm. To achieve reasonable gain for the entire desired bandwidth, the ground plane is trimmed. This technique also contributes to enhancing the key performance parameters of the antenna. The system impedance is assumed to be 50-ohm, and the overall size of the antenna is 16 mm × 12 mm × 0.787 mm. The dimensions of the proposed antenna elements are shown in Figure 1. They are A = 16 mm, B = 12 mm, C = 10.5 mm, D = 2 mm, E = 0.5 mm, F = 0.2 mm, G = 10 mm, and H = 12 mm. The radiating structure is composed of five circular petals and a circular hub. These petals are attached to the hub using rectangular branches. Each petal is separated by an angular distance of $d_{\theta }$ form its neighbour. For instance, the first petal is at $0^{\circ }$, the fifth at $180^{\circ }$, while others are $45^{\circ }$ apart from their neighbours. This whole system is connected to a 50 ohm transmission line. The length of the feed is 10 mm. In addition, the ground plane is trimmed, and a semi-circular shell is etched at the edge of the ground plane as shown in Figure 1b.

**Figure 2:**
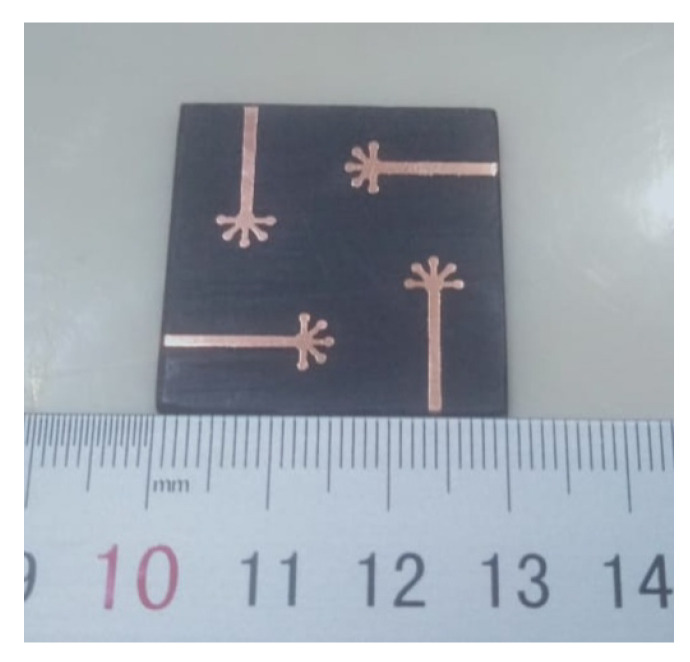
Fabricated prototype.

The proposed MIMO system is achieved by rotating a radiating element by $90^{\circ }$. This is done to achieve low mutual coupling and interference between the antenna elements. The MIMO system has a common ground plane with four semi-circular shells. The dimension of the proposed system is 30 mm × 30 mm. The system was simulated in Computer Simulation Technology (CST) software, and the results were verified by fabricating a prototype. The antenna was fabricated using the LPKF (Leiterplatten-Kopierfrasen) machine, as shown in Figure 2, and Anritsu Vector Network Analyzer (VNA) was used to measure the system.

## 3. Results and Discussion

In this section, the simulated and measured results of the proposed system are discussed. The system was modelled, simulated, and studied in Computer Simulation Technology (CST) 2020.

Before presenting the computed results, the effects and results of some parameters of the antenna design are discussed to understand the final design and its working principle.

**Figure 3:**
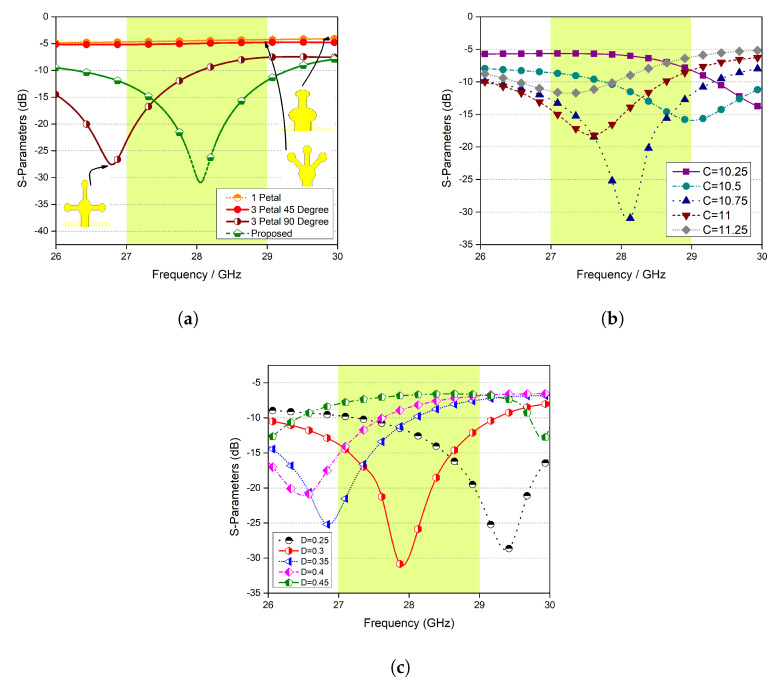
(**a**) Design evolution, (**b**) parametric modelling of feed length, and (**c**) parametric modelling of D.

Next, the evolution of the proposed design is discussed. First, a single petal was designed, and its response was observed. From Figure 3a, it can be seen that within the desired frequency range, the input signal is completely reflected, and hence, nothing is radiated. Secondly, three petals were designed $45^{\circ }$ apart; once again, nothing was radiated. For a shape consisting of three petals $90^{\circ }$ apart, a resonance was achieved close to the desired frequency range. Finally, a stem consisting of five petals was designed, and the desired results were obtained.

The effect of the length of the stem and the length of the stem of each petal was also studied, and the results are shown in Figure 3b,c, respectively. One can observe that these parameters changed the response of the proposed system drastically, as expected.

### 3.1. S-Parameters

**Figure 4:**
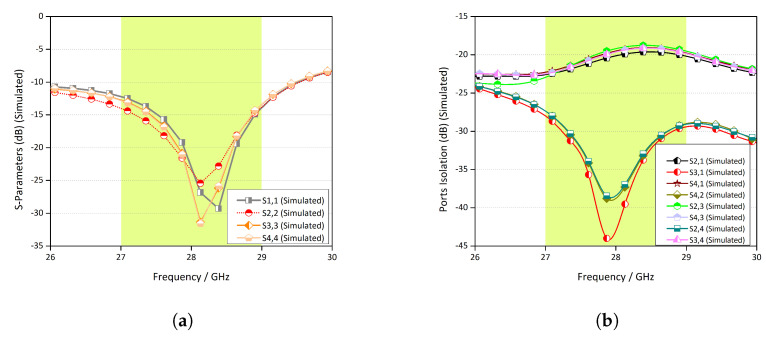

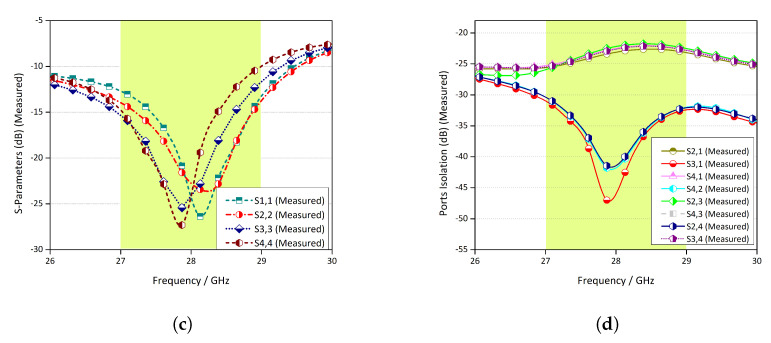
(**a**) Reflection coefficient of the proposed MIMO antenna configuration, (**b**) ports’ isolation between radiating elements, (**c**) measured S-parameters of the configuration, and (**d**) measured isolation between the antenna elements.

The simulated return loss of each port of the radiating elements is shown in Figure 4a. All the antenna elements exhibited almost the same behaviour. Figure 4b depicts the coupling between the radiating elements. Note that between any two given elements, the isolation is below −17 dB. This is because the antenna elements were arranged in such a manner to ensure a low mutual coupling between them. Figure 4c,d illustrates the measured reflection coefficients of the ports and coupling between the radiating elements.

**Figure 5:**
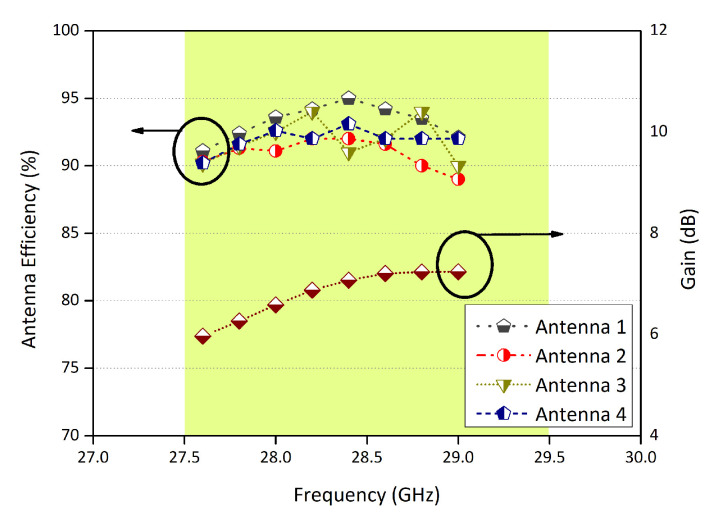
Efficiency and maximum gain of the proposed MIMO configuration.

The efficiency for each element of the proposed configuration is shown in Figure 5. The efficiencies range between 90% and 95%. Note that in Figure 5, the values on the y-axis on the right side are the maximum gain of the proposed configuration. It varies between 6 dB and 8 dB.

### 3.2. Radiation Pattern

**Figure 6:**
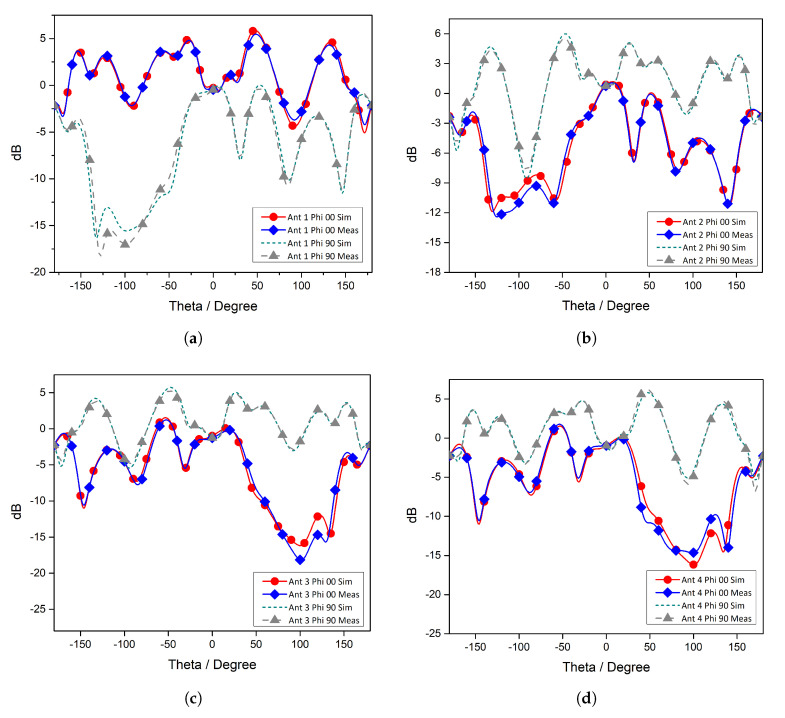
Simulated and measured radiation patterns: (**a**) Antenna Element-1, (**b**) Antenna Element-2, (**c**) Antenna Element-3, and (**d**) Antenna Element.

The behaviour of an antenna system in the far region is one of the important characteristics of a communication system. The radiation patterns of each antenna element for different azimuth angles and fixed elevation angles are shown in Figure 6. Figure 6a illustrates the comparison between the simulated and measured far-field pattern of Antenna 1 for $\phi =0^{\circ }$ and $\phi =90^{\circ }$. The results are in very good agreement. Note that the far-field pattern is almost circular in the xz-plane, while in the yz-plane, it is symmetric and directive with a maximum value at $60^{\circ }$. The radiation characteristics of Antenna 2 are depicted in Figure 6b. The radiation is approximately circular in the yz-plane, while in the xz-plane, the pattern is directive. The other radiating elements have similar far-field characteristics.

### 3.3. Surface Currents

**Figure 7:**
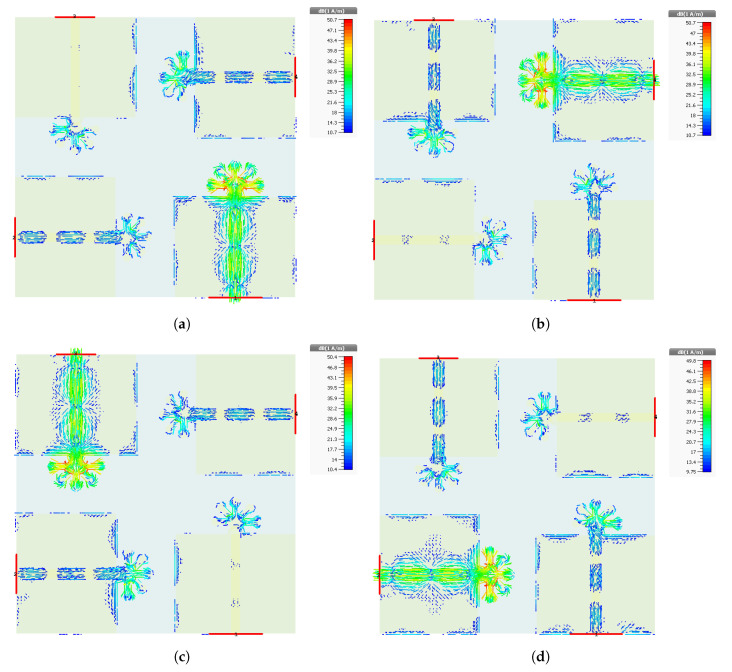
Surface current distribution at 28 GHz for (**a**) Antenna 1, (**b**) Antenna 2, (**c**) Antenna 3, and (**d**) Antenna 4.

The currents radiating in each antenna elements are shown. The surface current distribution of the proposed design is analysed at the frequency band of 28 GHz, as shown in Figure 7. The surface currents are analysed for each port, respectively. From the surface current distribution, it is observed that the current intensity is high across the stripes connecting the five circles and at these circles as well, which shows that the contribution of these five circles and connecting stripes at the top of the feed is more a mmWave frequency band achievement. Furthermore, the interference among the antenna elements is also negligible, as shown by the surface current distribution pattern in Figure 7. This is due to the arrangement of the antenna elements with a 90 degree shift with respect to each other.

## 4. MIMO Parameters

The MIMO parameters of the proposed antenna such as the co-relation coefficient, Mean Effective Gain (MEG), and Diversity Gain (DG) which determine the performance of MIMO Antenna system, are discussed in this section.

### 4.1. Envelope Correlation Coefficient

**Figure 8:**
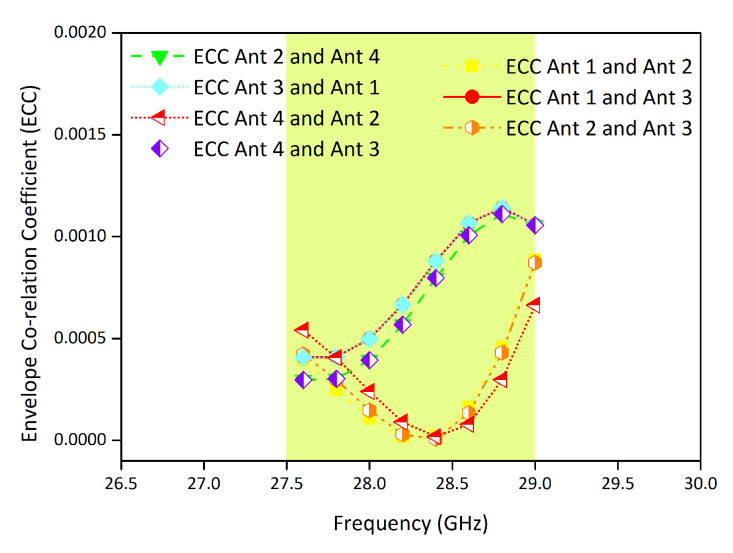
Envelope Correlation Coefficient (ECC) of the proposed MIMO antenna.

The envelope correlation coefficient is one of the important MIMO performance metrics, which describes the amount of coupling among the antenna elements of the MIMO configuration. For the proposed MIMO antenna, the ECC value is evaluated using the S-parameters method [26] and noticed below 0.0015 for the operating bandwidth, which demonstrates low mutual coupling among the antenna elements, as shown in Figure 8.

### 4.2. Mean Effective Gain

The ratio of the received and incident mean power is known as the Mean Effective Gain (MEG) of antenna. Ranging in between an adequate value of −3 and −12, the MEG is an important MIMO performance parameter of any MIMO antenna system. The MEGs of the proposed MIMO antenna system taken at three specific frequencies are shown in Table 1.

### 4.3. Diversity Gain

**Figure 9:**
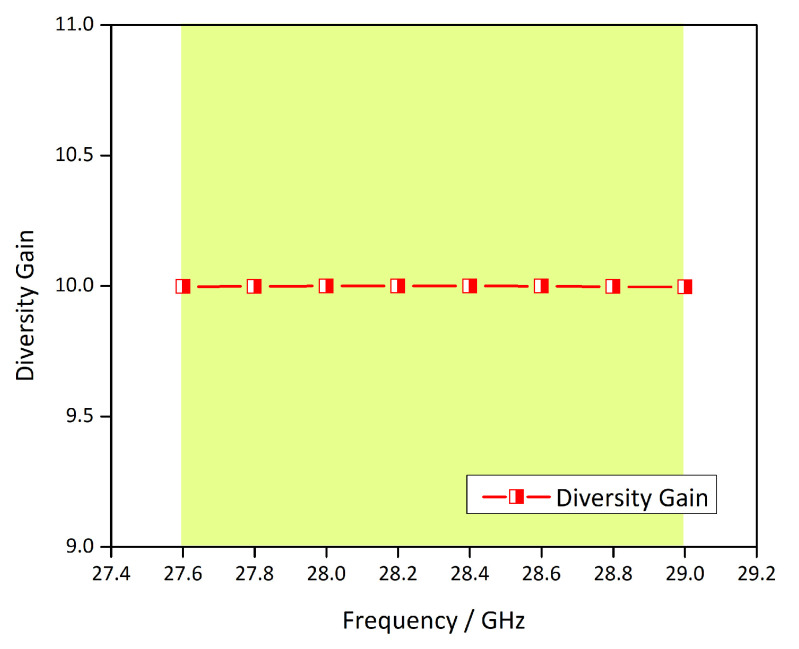
Diversity Gain (DG) of the proposed MIMO antenna.

The signals from multiple paths, received by the transmitter, are used to obtain the diversity gain. The greater levels of SNR are achieved by uncorrelated signals with a good reception of the signal. The transmission power reduction amount is described by the diversity gain without any loss in performance after the use of the diversity scheme for the antennas having the MIMO feature. The proposed MIMO antenna system’s DG is mentioned in Figure 9, derived by the equation mentioned in [26].

Table 2 shows the comparison of the published literature with the proposed MIMO antenna. From the comparison, it can be said that the proposed MIMO antenna is a promising candidate for future Ka-band 5G applications.

## 5. Conclusions

In this work, a flower-shaped four port MIMO antenna system is proposed. The configuration is modelled on a Rogers-5880 substrate. Different key performance parameters of the antenna and MIMO system are computed. A prototype is fabricated to verify the simulated results. It is found that the proposed system has very good return loss, low mutual coupling, low ECC, high gain, and high efficiency for the 5G 28 GHz band.

## Figures and Tables

**Table 1:** Calculated Mean Effective Gains (MEGs) of the MIMO antenna.

Frequency (GHz)	MEG1	MEG2	MEG3	MEG4
27.5	−3.45	−3.80	−3.77	−3.61
28	−3.76	−4.14	−3.85	−4.12
28.5	−3.57	−4.02	−5.13	−4.66
29	−5.02	−5.13	−4.60	−4.73

**Table 2:** Comparison table of the proposed MIMO antenna with the published literature.

References	Ports	Antenna Size in mm (L × W × H)	Isolation	Efficiency	Gain	ECC
[27]	1	25 × 15 × 0.762	–	99	5.9	–
[21]	6	75 × 110 × 0.76	−20	73	9.53	–
[28]	1	5 × 5 × 0.127	–	94	5.6	0.003
[29]	1	15 × 25 × 0.25	–	89	6.5	–
[30]	4	60 × 120 × 1.56	<19	80	<5	0.15
Proposed	4	25 × 15 × 0.787	−17	95	7.8	0.0001
